# Mastectomy with Reconstruction Including Robotic Endoscopic Surgery (MARRES): a prospective cohort study of the Korea Robot-Endoscopy Minimal Access Breast Surgery Study Group (KoREa-BSG) and Korean Breast Cancer Study Group (KBCSG)

**DOI:** 10.1186/s12885-023-10978-0

**Published:** 2023-06-21

**Authors:** Jai Min Ryu, Jeea Lee, Jeeyeon Lee, BeomSeok Ko, Joo Heung Kim, Hyukjai Shin, Hyung Seok Park, Korea Robot-endoscopy Minimal Access Breast Surgery Study Group (KoREa-BSG)

**Affiliations:** 1 Division of Breast Surgery, Department of Surgery, Samsung Medical Center, Sungkyunkwan University School of Medicine, Seoul, Korea; 2 Department of Surgery, Uijeongbu Eulji Medical Center, Eulji University, Gyeonggi-Do, Korea; 3 Department of Surgery, Graduate School of Medicine, Yonsei University College of Medicine, Seoul, Korea; 4 Department of Surgery, Kyungpook National University School of Medicine, Daegu, Korea; 5 Department of Surgery, University of Ulsan College of Medicine, Asan Medical Center, Seoul, Korea; 6 Department of Surgery, Yongin Severance Hospital, Yonsei University College of Medicine, Yongin, Gyeonggi Korea; 7 Department of Surgery, Myongji Hospital, Hanyang University Medical Center, Goyang, Korea; 8 Department of Surgery, Yonsei University College of Medicine, 50-1, Yonsei-Ro, Seodaemun-Gu, Seoul, 03722 Republic of Korea

**Keywords:** Breast neoplasms, Conventional nipple-sparing mastectomy, Endoscopic nipple-sparing mastectomy, Germline *BRCA1/2* mutation, Robotic-assisted nipple-sparing mastectomy, Minimally invasive procedure, Immediate breast reconstruction

## Abstract

**Background:**

Robotic nipple-sparing mastectomy (RNSM) has emerged as a new treatment option for breast cancer and risk-reducing mastectomy (RRM) for women who have a high risk of pathogenic variants. Even though several studies have reported that RNSM is a feasible procedure, some argue that it should only be performed by specialized surgeons, and data on oncologic outcomes and patient-reported outcomes (PROs) are limited. Recently, the United States Food and Drug Administration and several surgeons warned that robotic breast surgery should be performed only by specialized surgeons and recommended that the benefits, risks, and alternatives of all available treatment options be discussed with patients so they can make informed treatment decisions. The Korea Robot-Endoscopy Minimal Access Breast Surgery Study Group (KoREa-BSG) has been established to evaluate, standardize, and teach this state-of-the-art procedure. We have designed a multicenter prospective cohort study entitled Mastectomy with Reconstruction Including Robot Endoscopic Surgery (MARRES) to report surgical, PRO, and oncologic outcomes.

**Methods:**

MARRES is a multi-institution cohort study prospectively collecting data from patients undergoing mastectomy and reconstruction. The patient inclusion criteria are adult women older than 19 with breast cancer or a high risk of breast cancer (patients with *BRCA1/2*, *TP53*, *PALB2* mutations, etc.), who have scheduled therapeutic or RRM and want immediate reconstruction. Surgical outcomes, including pre- and postoperative photos, oncologic outcomes, cost-effectiveness, and PRO, are collected. The primary endpoints are postoperative complication rates within 30 postoperative days and the Clavien-Dindo grade of postoperative complications within 180 postoperative days. The secondary endpoints are 5-year postoperative recurrence-free survival and cancer incidence rate (for those who underwent RRM), patient satisfaction with reconstruction expectations preoperative (baseline) and results within 6 to 12 postoperative months, surgeon satisfaction with postoperative results in 6 postoperative months, and cost-effectiveness of the definitive surgery. Patient recruitment will be completed in April 2025, and the target number of enrolled patients is 2000.

**Discussion:**

This study will provide evidence about the surgical outcomes, oncologic outcomes, and patient satisfaction with RNSM and endoscopic nipple-sparing mastectomy (NSM), compared with conventional NSM.

**Trial registration:**

ClinicalTrials.gov Identifier NCT04585074. Registered April 8, 2020.

## Background

Since Toth and Lappert first described a skin-sparing mastectomy (SSM) procedure, similar oncologic outcomes and better patient satisfaction and quality of life (QOL) have been reported for it compared with conventional mastectomy (CM) [1–3]. Immediate breast reconstruction (IBR) is facilitated by preserving the skin envelope at the time of mastectomy, and the success of SSM has paved the way for nipple-sparing mastectomy (NSM). Because NSM has shown better patient satisfaction and cosmetic results than SSM, with comparable oncologic outcomes, it has become popular [4, 5]. NSM with IBR has become widespread as indications have expanded. Increased *BRCA1/2* genetic testing and public awareness have led to a rise in risk-reducing mastectomy (RRM), and there is increased interest in QOL after mastectomy [6, 7].

In NSM, various kinds of skin incisions can be selected [8–10]. To prevent visible scarring and minimize the incision size, endoscopic NSM (ENSM) was developed more than 20 years ago [11–13]. However, ENSM has several limitations: (1) complex devices are needed, (2) the approaches are difficult for endoscopic devices, especially in the medial part of the breast, and (3) it is labor-intensive for surgeons and assistants, especially for large and ptotic breasts. Therefore, ENSM has been performed only by specialized surgeons in East Asia.

Robots are used for various surgeries, including those for malignant diseases. Since the first reports of robotic nipple-sparing mastectomy (RNSM) by Toesca et al. and Park et al. in Korea, RNSM has been performed in selected patients and *BRCA1/2* carriers [14, 15]. Even though several studies have shown that RNSM is a feasible procedure, it is performed by only a few specialized surgeons [16]. Furthermore, few data have been reported about its oncologic outcomes.

Recently, the United States Food and Drug Administration warned that robotic breast surgery should be performed only by specialized surgeons and recommended that the benefits, risks, and alternatives of all available treatment options be discussed with patients so they could make informed treatment decisions. Previous articles have also recommended that randomized controlled trials be conducted to assess the surgical safety, patient reported outcomes (PROs), and oncologic outcomes of RNSM and thus evaluate its clinical role in treating *BRCA1/2* carriers and patients with breast cancer [17, 18].

The Korea Robot-Endoscopy Minimal Access Breast Surgery Study Group (KoREa-BSG) has been established to evaluate, standardize, and teach this state-of-the-art procedure. Previously, the KoREa-BSG retrospectively analyzed the initial experiences of surgeons and showed that RNSM is technically feasible and reliable, with a short learning curve [19]. Therefore, to obtain a high level of evidence, we designed a multicenter prospective exploratory cohort study entitled Mastectomy with Reconstruction Including Robotic Endoscopic Surgery (MARRES) to collect data on surgical outcomes, PROs, and oncologic outcomes (Protocol serial numbers: KoREa-BSG 03 and KBCSG-23).

### Evidence for immediate reconstruction

#### Guidelines and reviews for conventional NSM (CNSM) with IBR

The first NSM was described by Freeman in 1962 but only for RRM [20]. Subcutaneous mastectomy for patients with primary breast cancer was first reported by Hinton et al. in 1984 and showed rates of local recurrence and survival comparable to those with CM [21]. The use of NSM for women with breast cancer has been justified by demonstrating its oncologic and surgical safety. Previous studies confirmed acceptable locoregional recurrence, disease-free survival, and overall survival (OS) rates following NSM [4, 22–27]. The surgical outcomes of NSM, including rates of nipple necrosis and overall postoperative complications, were acceptable in previous reports [4, 22, 23]. The procedure’s advantages in terms of better aesthetic outcomes, improved patient satisfaction, and psychosexual benefits have also been investigated [28–30].

The National Comprehensive Cancer Network (NCCN) guideline comments that SSM showed similar oncologic outcome compared to CM [31]. However, SSM should be performed by an experienced breast surgeon. Similarly, NSM is comparable oncologic outcome to SSM, if cases are appropriately selected. NSM can be performed for any tumor size independent of axillary status, especially for early breast cancer, and for ductal carcinoma in situ and RRM, but having cT4b and cT4c breast cancers with skin involvement or skin edema, and clinical signs of nipple involvement and any R1 resection at the nipple margin should be a contraindication.

#### Guidelines and reviews for ENSM with IBR

Due to aesthetic concerns about the breast, breast surgery has been developed to satisfy both oncologic safety and cosmetic needs [13]. Endoscopic procedures have been used for various breast surgeries, including augmentation, mastectomy, excision, biopsy, capsulectomy, and reconstruction [32].

Endoscopic-assisted breast surgery (EABS) was introduced in the late 1990s to optimize the aesthetic effects by using a minimal incision in an inconspicuous location for benign breast disease or breast cancer [33]. Previous studies reported excellent patient satisfaction on patient-reported questionnaires after ENSM [34–36]. Additionally, endoscopy could offer better visualization with light handle retractions through a small incision and allow similar oncologic outcomes compared to CNSM [13, 37]. Indications and contraindications for EABS are similar to CNSM. Although ENSM is considered a safe and feasible procedure that leaves a relatively inconspicuous incision in patients with breast cancer [38], endoscopic techniques for breast surgery have not been widely performed because of the technical challenges with using rigid instruments [39].

#### Guidelines and reviews for RNSM with IBR

Since the first robotic surgical system was introduced in 2000, robotic surgical systems have spread widely into various surgical fields because of the comfort and ergonomic movement they allow, with magnified and high-resolution vision [40]. Robotic breast surgery has also been developed and investigated by several pioneers to establish clinical evidence for this new technique (Tables 1 and 2) [19, 41–47].

**Table 1:** General information on previous studies about RNSM

Study	Year	Study interval	Aim	Design	No. of patients (procedures)	Indication (*n*, %)
Ryu et al. [19]	2020	Nov 2016–Jan 2020	To report the early experience of RNSM with IBR in the KoREa-BSG	Retrospective case series, multicenter	73 (82)	Therapeutic (75, 91.5%)Risk-reducing (7, 8.5%)
Lee et al. [41]	2021	Nov 2016–Jan 2019	To directly compare surgical outcomes between CNSM and RNSM	Retrospective case–control comparison study, single center	CNSM 270 RNSM 41	Therapeutic (34, 83%) Risk-reducing (7, 17.1%)
Toesca et al. [42]	2019	June 2014–Jan 2019	To present and discuss perioperative surgical outcomes and early oncologic follow-up data on consecutive patients undergoing RNSM	Prospective case series, single center	73 (94)	Invasive breast cancer (39, 41.5%) DCIS (21, 223%) Risk-reducing (34, 36.2%)
Sanson et al. [43]	2018	Nov 2015–Jan 2020	To report the feasibility of RNSM with a large series of 138 procedures	Prospective, single center	79 (138)	Prophylactic (75%) Therapeutic (25%)
Lai et al. [44]	2020	July 2011–Sep 2019	To critically compare RNSM vs. CNSM procedures from different aspects (clinical outcomes, patient-reported aesthetic results, and medical costs) in the management of patients with breast cancer	Retrospective case–control comparison study, single center	CNSM 62 RNSM 54	Therapeutic
Houvenaeghel et al. [45]	2021	Nov 2016–Mar 2020	To compare RNSM and CNSM in terms of the breast complication rate (main objective) and hospital stay, duration of surgery, cost evaluation, and patient satisfaction (secondary objectives)	Prospective case–control comparison study, single center	CNSM 142 RNSM 87	Primary (70, 80.5%) Local recurrence (10, 11.5%) Prophylactic (7, 8.0%)
Loh et al. [46]	2020	April 2018–Jan 2020	To report the use of RNSM in patients with breast cancer and analyze the learning curve of one surgeon in a single medical center	Retrospective single center	78 (85)	Therapeutic
Kuo et al. [47]	2019	N/A	To report the combination of robotic mastectomy and immediate microsurgical free-flap reconstruction and exploit its oncologic and aesthetic advantages	Case series, single center	3	Therapeutic

*CNSM* conventional nipple-sparing mastectomy, *IBR* immediate breast reconstruction, *KoREa-BSG* Korea Robot-Endoscopy Minimal Access Breast Surgery Study Group, *RNSM* robotic nipple-sparing mastectomy

**Table 2:** Summary of ongoing studies of RNSM

Identifier	Sample size	Randomization	Indication	Intervention	Postoperative outcomes	Oncologic outcomes	PRO	Country of origin
NCT04585074 (current study)	2000	No	Women with breast cancer High-risk women	RNSM + ENSM CNSM	Yes	Yes	Yes	South Korea
NCT04108117	300	No	Women with breast cancer High-risk women	RNSM CNSM	Yes	Yes	No	South Korea
NCT03440398	82	Yes	Women with breast cancer High-risk women	RNSM CNSM	Yes	Yes	Yes	Italy
NCT03892980	145	No	High-risk women	RNSM	Yes	No	No	United States
NCT04537312	20	No	Women with breast cancer High-risk women	RNSM	Yes	No	Yes	United States
NCT04457167	480	No	Women with breast cancer High-risk women	RNSM CNSM	Yes	No	No	France
NCT04151368	30	No	Women with breast cancer High-risk women	RNSM	Yes	Yes	Yes	Canada
NCT04037852	180	No	Women with breast cancer	RNSM CNSM or ENSM	Yes	Yes	Yes	Taiwan

*CNSM* conventional nipple-sparing mastectomy, *ENSM* endoscopic nipple-sparing mastectomy, *PRO* Patient-reported outcome, *RNSM* robot-assisted nipple-sparing mastectomy

No intraoperative or postoperative mortality caused by RNSM has been reported and only one case of open conversion experienced in the literature [16]. The rates of postoperative complications have primarily been acceptable [16, 41, 42, 48]. In terms of oncologic safety, a few reports have indicated a favorable incidence of margin positivity and no locoregional recurrence within short-term follow-up [42, 47, 48]. The innovative robotic technique in breast surgery has been presented as a safe and feasible surgical procedure that is not inferior to the conventional methods in terms of early oncological outcomes [42].

As RNSM attracted attention and became the focus of more research, standardized RNSM guidelines were developed by a representative panel of 10 international experts in 2019 [49]. Previous studies have suggested that an indication and contraindication for RNSM is similar to CNSM [48–50].

## Methods/Design

**Fig. 1:**
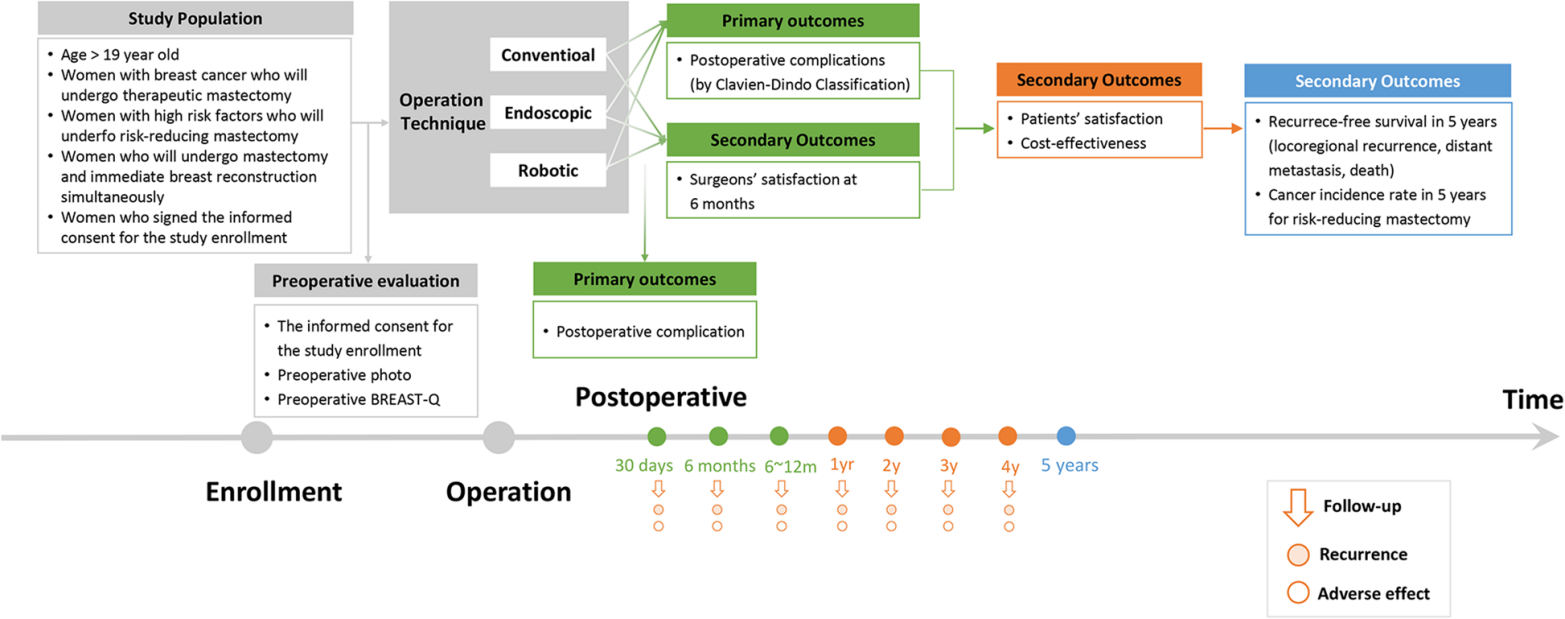
Flowchart of the prospective cohort study

The MARRES study is a multi-institution cohort study being conducted by KoREa-BSG. It is prospectively collecting clinical data from patients undergoing mastectomy and reconstruction in academic hospitals in Korea (Fig. 1). The version 1.71 protocol for this study was approved on June 27, 2022. The principal investigator (PI) will periodically review and evaluate study conduct and progress according to the study protocol; data collection status for participant safety; and the quality, consistency, security, and accessibility of the accumulated data at regular intervals during the study. The protocol and study progress will be audited annually according to the policy of each Institutional Review Board. The PI and sub-PIs meet to communicate every one or two months. Only the PI and sub-PIs will have access to the final trial dataset, but the dataset could be shared with the permission of the PI upon reasonable request. We will publish the results of the dataset in a medical journal.

The inclusion criteria for the patients are as follows: adult women older than 19 with breast cancer or a high risk of breast cancer (patients with *BRCA1/2*, *TP53*, *PALB2* mutations, etc.) who are scheduled for a therapeutic or risk reducing mastectomy and want immediate reconstruction. The PI or a physician delegated by the PI provides information about the study and an adequate opportunity to consider all options to all potential trial participants and obtains signed informed consent forms before enrollment. Patients who plan breast-conserving surgery or who are not candidates for IBR will be excluded. The target number of enrolled patients is 2000.

Patients' clinicopathological factors, including height and weight, will be collected, along with data on surgical results, including photographic and oncological results and cost-effectiveness and patient satisfaction data. The basic characteristics, photographs, and satisfaction of patients will be collected preoperatively using the Breast-Q survey. Patient data will be collected within 6 months of surgery, including clinicopathological factors, surgery results (drainage amount, removal date, and complications), postoperative recovery evaluations, complications and adverse reactions, and cost. Between 6 months and 1 year after surgery, the results of surgery, recurrence, a satisfaction survey, intraoperative console video, and postoperative photographs will be collected. Every 12 months thereafter, data on adjuvant therapy (chemotherapy, radiation therapy, targeted therapy, and endocrine therapy), surgical results, recurrence, adverse events, and other unintended effects of the interventions will be investigated and collected until the end of the study period.

Personal information that can identify the subjects will be kept confidential even after the publication of the study. After enrollment, each participant’s name will be encrypted and replaced with a participant number. The datasets collected from each institution will be accumulated and stored in a designated computer on a central server. Access to that database is restricted to authorized researchers.

An interim analysis will be done after the recruitment of subjects is completed, no later than the 3rd year after the start of data collection. The PI will access those interim results and report them to the all sub-PIs. At that time, the PI and sub-PIs will determine whether the trial should be terminated.

Follow-up observation and data collection of the subjects will continue for 4–8 years after enrollment, and then the final analysis will be conducted. All patients will be guided to complete follow-up evaluations indicating recurrences and survival for at least 5 years.

### Timeline

- Actual Study Start Date: April 8, 2020
- Estimated Primary Completion Date: April 7, 2025
- Estimated Study Completion Date: April 7, 2030

### Study population

Intervention details.

#### Procedure: Robotic nipple-sparing mastectomy

Patients undergoing RNSM and IBR are enrolled in this arm. RNSM should be performed using a robotic surgical system (da Vinci S, Si, X, Xi, or SP). Axillary or lateral incisions are used for this procedure.

Other names: Robot-assisted nipple-sparing mastectomy, robot mastectomy, robotic mastectomy, hybrid robotic nipple-sparing mastectomy, robot-assisted nipple-areolar complex, and skin-sparing mastectomy.

#### Procedure: Endoscopic nipple-sparing mastectomy

Patients undergoing ENSM and IBR are enrolled in this arm. ENSM should be performed using endoscopic tools for mastectomies [51].

Other names: Endoscopy-assisted nipple-sparing mastectomy, endoscopic-assisted nipple-sparing mastectomy, endoscopic nipple-sparing mastectomy, endoscopic subcutaneous mastectomy, video-assisted nipple-sparing mastectomy, endoscopic video-assisted breast surgery, videoendoscopic nipple-sparing mastectomy.

#### Procedure: Conventional mastectomy (including nipple-sparing mastectomy, skin-sparing mastectomy)

Patients undergoing a CM and immediate reconstruction are enrolled in this arm. CM should not be performed using a robotic or endoscopic surgical system. Any incisions can be used for this procedure. CM also includes NSM and SSM.

Other names: Total mastectomy, mastectomy, nipple-sparing mastectomy, skin-sparing mastectomy.

### Endpoints

#### Primary endpoint

Postoperative complication rates within 30 postoperative days: Postoperative complication rates are calculated as the total number of postoperative complication cases per total operation cases.

Clavien-Dindo grade of postoperative complications in 180 postoperative days: The Clavien-Dindo grade of postoperative complications is evaluated. Only grade III or higher postoperative complications are used for this analysis.

#### Secondary endpoints

Recurrence-free survival (RFS) in 5 postoperative years: RFS events include locoregional recurrence, distant recurrence, and death by any cause. Contralateral breast cancer and a second primary malignancy are considered to be censored data.

Cancer incidence rate in 5 postoperative years: Cancer incidence rate for those who underwent a prophylactic mastectomy.

Patient satisfaction with reconstruction results preoperative (baseline) and 6 to 12 postoperative months: Reconstruction module with pre- and postoperative scales for satisfaction with the abdomen, as assessed by BREAST-Q version 2.0. (This scale should be completed only by patients who have had reconstruction using a TRAM flap or DIEP flap. Otherwise, it is skipped.) Satisfaction with the back, as assessed by BREAST-Q version 2.0. (This scale should only be completed by patients who have had reconstruction using an LD flap. Otherwise, it is skipped.) Satisfaction with implants, as assessed by BREAST-Q version 2.0. (This scale should only be completed by patients who have had reconstruction using implants. Otherwise, it is skipped.) In all scales, higher scores reflect better outcomes.

Surgeon's satisfaction with surgery within 6 postoperative months: Assessed using scoring criteria for cosmetic assessment [52], response options (overall symmetry, postoperative scar, NAC symmetry, etc.), and range (0–10). Higher scores reflect better outcomes.

Evaluation of the cost-effectiveness of the definitive surgery according to the surgical method: Assessed by conducting a patient’s survey 6 months to 1 year after the last surgery. This evaluation uses the EuroQol five-dimension scale, Korean version questionnaire. In all scales, higher scores reflect better outcomes.

### Inclusion & exclusion criteria

Inclusion Criteria:

- Female patients older than 19
- Patients with breast cancer or a high risk of breast cancer (*BRCA1/2*, *TP53*, *PALB2* mutations, etc.)
- Patients scheduled for a therapeutic or risk reducing mastectomy (including conventional, skin-sparing, and areolar-conserving mastectomies)
- Patients who want immediate reconstruction
- Patients who provide written consent to participate in the study

Exclusion Criteria:

- Patients scheduled for a breast-conserving surgery
- Patients who do not want immediate reconstruction during mastectomy
- Patients who undergo a different procedure on the other side breast simultaneously

Surgeon Inclusion Criteria:

- Surgeons who are members of KoREa-BSG
- Surgeons who participated in an education program for RNSM as an operator more than once

### Stopping criteria

Patients are free to withdraw from the study at any point without limitation. All data collected before the withdrawal will be included. After a withdrawal, no additional data will be collected from that patient. If the PI determines that it is inappropriate to continue the clinical trial, some part or the entire study could be stopped.

### Sample size determination

We determined the sample size for this study according to the real clinical experience of the PI, without statistical calculation, to explore the safety and effectiveness of the robotic surgical system and derive clinical outcomes from the procedures. The main institution performed 4038 cases of breast surgery and 746 cases of mastectomy and IBR (18.5%) between 2016 and 2018, including 86 RRMs (86/746, 11.5%). The iBRA study reported the uni- or bilateral RRMs accounted for 34.8% of mastectomies [53]. The mean value of the RRM rate between the main institution and the iBRA study was 23.15%. We estimated an approximate sample size of 11.5% for the main institution and 23.15% for the other institutions.Planned enrollment is 2000 total patients across the conventional, endoscopic, and robotic groups.

### Pre-specified subgroup analysis

Because of the long duration and expensive cost of a randomized trial, we will extract random patients from our prospective cohort and conduct subgroup analyses as a randomized registry trial. In a previous study, the rates of grade III complications in the reference group and the robotic group were 34.8% and 17.2%, respectively. For the comparison of complication rates among different surgical methods, the required sample size was calculated as 112 (1:1 random match) according to the method used in a randomized registry trial previously introduced to detect a non-inferiority margin difference, which achieved 80% power between the group proportions of 0.0500. The robotic group proportion is assumed to be 39.8% under the null hypothesis of inferiority. The power was computed for the case in which the actual robotic group proportion is 17.1%. The test statistic used is the one-sided Z test (unpooled). The significance level of the test was targeted at 0.0250. The actual significance level achieved by this design is 0.0265.

### Statistical analyses

We have categorized three study sets: Conventional vs. endoscopic, conventional vs. robotic, and conventional vs. endoscopic and robotic. Additionally, subgroup analyses of a randomized registry trial will be conducted to compare the surgical and oncologic outcomes between the conventional and minimally invasive (endoscopic and robotic) groups. Primary and some secondary outcomes (postoperative complications, the satisfaction of patients and surgeons, and the cost-effectiveness evaluation) and other categorical variables will be examined by the chi-square test or Fisher's exact test. Continuous variables will be examined by t-testing or ANOVA and Mann–Whitney testing or Kruskal–Wallis testing, if needed. The other secondary outcomes (RFS, OS, and cancer incidence rates in 5 years) will be examined using Kaplan–Meier plots and log-rank testing. No imputation will be performed for subjects who have missing data due to dropping out of the study. Data relating to major protocol non-adherence due to patients dropping out of the study will be excluded from the analysis population.

## Discussion

This study will provide evidence about the surgical outcomes, oncologic outcomes, and patient satisfaction with RNSM and ENSM, compared with CM.

This prospective cohort study has some advantages over previous retrospective studies conducted in a single center or with a single surgeon. The sample size of the study is larger than previously published studies of RNSM. Additionally, our protocol will collect large-scale PRO data about cosmesis after RNSM. The participation of many surgeons from multiple institutions will allow us to observe the effects of different surgeons or institutions on the clinical outcomes of the surgical procedures. In Korea, an increasing number of breast surgeons has been performing RNSM because of active educational programs and systematic research activities. Therefore, the involvement of many skilled surgeons will produce accurate clinical outcomes for the procedures. However, because this is not a randomized controlled trial, selection bias might still occur in determining the surgical modality for each subject. To overcome that limitation and secure the surgical and oncologic outcomes from a new, innovative surgical procedure, we are planning a randomized controlled trial for the near future.

Through this study, we wish to confirm that minimally invasive breast surgery can be considered as a standard treatment for women with breast cancer and as a preventive treatment for women with a high risk of pathogenic variants.

## Data Availability

The datasets used and/or analyzed during the current study are available from the corresponding author upon reasonable request.

## Abbreviations

CM: Conventional mastectomy
CNSM: Conventional nipple-sparing mastectomy
DIEP: Deep inferior epigastric perforators
DTI: Direct-to-implant
EABS: Endoscopic-assisted breast surgery
ENSM: Endoscopic nipple-sparing mastectomy
IBR: Immediate breast reconstruction
KoREa-BSG: Korea Robot-Endoscopy Minimal Access Breast Surgery Study Group
LD: Latissimus dorsi
MARRES: Mastectomy with Reconstruction Including Robot Endoscopic Surgery
NAC: Nipple-areolar complex
NSM: Nipple-sparing mastectomy
OS: Overall survival
OPBC: Oncoplastic Breast Consortium
PI: Principal investigator
PRO: Patient-reported outcomes
QOL: Quality of life
RFS: Recurrence-free survival
RNSM: Robotic nipple-sparing mastectomy
RRM: Risk-reducing mastectomy
SSM: Skin-sparing mastectomy
TRAM: Transverse abdominis rectus muscle
